# Factors Influencing the Severity and Progression of Respiratory Muscle Dysfunction in Myotonic Dystrophy Type 1

**DOI:** 10.3389/fneur.2021.658532

**Published:** 2021-04-13

**Authors:** Leigh Hartog, Jing Zhao, Jerry Reynolds, Gabrielle Brokamp, Ferdinand Vilson, W. David Arnold, Samantha LoRusso

**Affiliations:** ^1^Department of Neurology, The Ohio State University College of Medicine, Columbus, OH, United States; ^2^Department of Biomedical Informatics, The Ohio State University College of Medicine, Columbus, OH, United States; ^3^Department of Neurology, Ohio State University Medical Center, Columbus, OH, United States

**Keywords:** myotonic dystrophy, respiratory, impairment, PFT, FVC

## Abstract

Respiratory complications are the most common cause of death among patients with Myotonic Dystrophy type 1 (DM1), but the natural history of respiratory decline in DM1 patients is incompletely characterized and few predictors of the progression of respiratory dysfunction have been identified. To identify factors influencing the progression of respiratory dysfunction electronic medical records from 110 adult patients diagnosed with DM1 were reviewed along with data for respiratory symptoms and pulmonary function obtained from routine respiratory therapist clinical evaluations. At baseline, 70.9% had evidence of restrictive respiratory impairment. We examined various parameters of respiratory functional status, and found FVC (% predicted) correlated best with other measures of disease severity. Annual change in FVC was −1.42 (std error = 0.381). Greater CTG repeat size, higher MIRS rating, and longer disease duration were all correlated with lower baseline FVC but not with annual rate of change. Wide variability in clinical phenotype made determination of disease measures directly related to respiratory functional decline challenging.

## Highlights

- Forced vital capacity (FVC) as a measure of respiratory impairment in Myotonic Dystrophy type 1.
- FVC impairment is associated with greater CTG repeat size, higher MIRS rating, or longer disease duration.
- Slowly declining restrictive respiratory impairment in Myotonic Dystrophy type 1.
- Rate of FVC decline in Myotonic Dystrophy type 1 not associated with greater CTG repeat size, higher MIRS rating, or longer disease duration.

## Introduction

As the most common muscular dystrophy in adults, myotonic dystrophy type 1 (DM1) affects about 1 in 8,000 individuals (1). Muscle weakness, atrophy, myotonia are associated with prominent multisystem involvement including central nervous system dysfunction, endocrinopathies, and cardiac conduction abnormalities (2). Respiratory failure is the most common cause of death in DM1 patients and conversely DM1 is a likely under recognized cause of respiratory failure in the undifferentiated patient presenting with respiratory failure (3–5). This is likely due to a combination of central respiratory drive dysfunction and the effects of skeletal muscle weakness along with upper airway muscle dysfunction leading to obstructive sleep apnea and aspiration (3, 4). Restrictive ventilatory patterns with impaired forced vital capacity (FVC), vital capacity (VC), max inspiratory pressure (MIP), and max expiratory pressure (MEP) and associated chronic hypercapnia and sleep disordered breathing are common in DM1 (6). While it's well-established that general muscle impairment is more severe with higher CTG repeat size and progresses throughout disease duration, increased respiratory dysfunction has been inconsistently correlated with higher CTG repeat sizes and skeletal muscle weakness without consensus (4, 6–8). Furthermore, the natural history of respiratory functional decline in DM1 is incompletely understood, and patient characteristics that may be predictive of this decline are unknown.

One recent retrospective study found that there were differences between groups of DM1 patients in the time course of respiratory function decline, such that patients who developed restrictive lung disease during the follow up period had a faster annual decline in percent predicted total lung capacity (TLC) than those who began the study with restrictive lung disease (8). Other studies have reported declines in spirometry over time but have not elucidated factors related to a fast or slow decline (9–11). Gaining an understanding of the natural history of lung dysfunction in the DM1 patient population will aid in risk assessment, understanding of pathogenesis, and development of appropriate outcome measures and treatments for future therapeutic trials.

We performed a retrospective study of DM1 patients seen in the Neuromuscular Clinic at the Ohio State University Wexner Medical Center. Aims of our study were to (I) examine the natural history of respiratory dysfunction, (II) assess the associations between respiratory muscle dysfunction and other patient characteristics, and (III) identify factors that influence progression of respiratory dysfunction.

## Materials and Methods

### Patients and Data Collection

Electronic medical records were reviewed from patients 18 years of age or older diagnosed with DM1 seen in the Neurology Department at The Ohio State University Wexner Medical Center between March 2009 and July 2019. Demographics, height, weight, clinical examination, respiratory symptoms, and pulmonary function tests (PFTs) were recorded for each patient at every nearest 6-month interval when data was available. Severity of weakness was graded from 0 to 5 using manual muscle testing by a neuromuscular specialist and each patient was assigned a severity grade based on the muscular impairment rating scale (MIRS) that is commonly used to quantify severity of muscle weakness in DM1 (12).

Respiratory symptoms and pulmonary function were assessed during routine clinical evaluations by a single respiratory therapist in the neuromuscular clinic. Patients were specifically asked about shortness of breath, dyspnea on exertion, orthopnea, night sweats, morning headaches, and, when applicable, compliance with non-invasive ventilation. Routine pulmonary testing included measurements of oxygen saturation (SpO_2_), end-tidal CO_2_ (EtCO_2_), total hemoglobin (SpHb), forced vital capacity (FVC), forced expiratory volume (FEV1), FVC/FEV1, peak expiratory flow (PEF), vital capacity (VC), maximal inspiratory pressure (MIP), and maximal expiratory pressure (MEP). Analyses were focused on FVC as an index of ventilatory muscle function. PFT severity was rated according to the Knudson scale with FVC % predicted normal if >79%, mild restriction if 70–79%, moderate restriction if 50–69%, and severe restriction if less than 50%. Any patients with comorbid pulmonary diagnoses (i.e., asthma, pulmonary embolism, lung cancer, lung lesion or mass, alpha-1-antitrypsin deficiency, etc.) or exclusively obstructive impairments noted on pulmonary function tests were excluded. Ethical standards were adhered to in accordance with The Ohio State University's Institutional Review Board.

### Statistical Analysis

Factors influencing respiratory dysfunction at baseline were investigated with Pearson correlations. The relationships of specific respiratory symptoms and respiratory dysfunction at baseline were investigated with Kruskal-Wallis tests. Kruskal-Wallis analyses were used to compare the annual rate of change of FVC and EtCO_2_ across CTG, age of onset, MIRS groups, and PFT severity; if significant, Wilcoxon tests were used for pairwise comparison. Correlation between annual rate of change of FVC and EtCO_2_ vs. BMI were calculated using Kendall's tau correlation. Multiple regression was used to model changes in FVC (% predicted) over time using the covariates of CTG repeat length, MIRS score, age at time obtained, and time since baseline. Restricted cubic spline functions with 3 default knots were used to allow CTG, age, and time since baseline to act smoothly but non-linearly. Generalized least squares was used in estimating the model coefficients while accounting for the temporal correlation between residuals. A generalized least squares analysis was used to calculate a slope estimate for the annual change of FVC (% predicted) to quantify change in FVC over time. Comparison of rate of change of FVC (% predicted) between patients recommended and compliant vs. non-compliant with NIV was made with multiple regression analyses fitted using generalized least squares.

## Results

### Populations Characteristics and Factors Associated With Respiratory Dysfunction at Baseline

**Figure 1:**
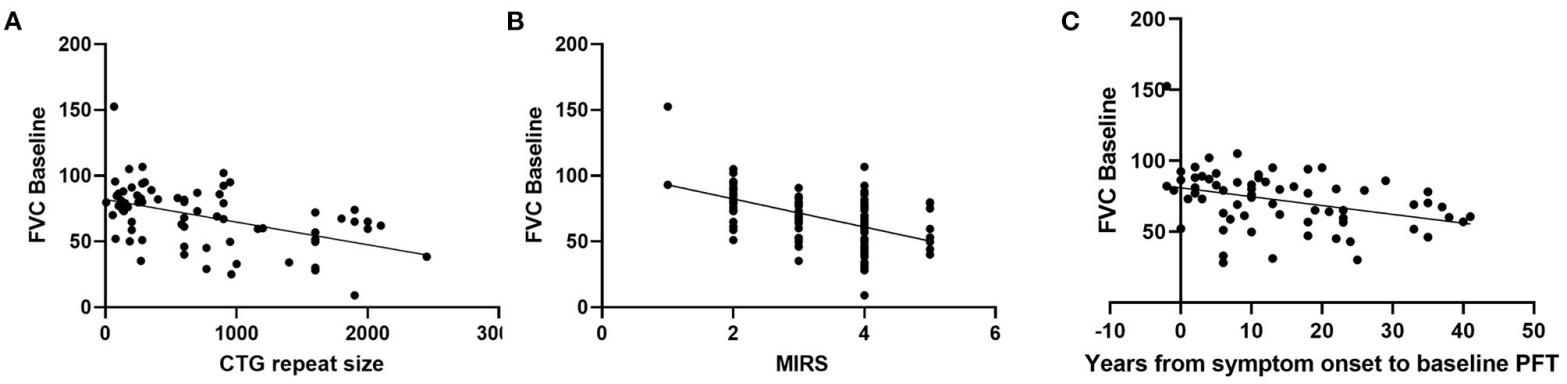
Relationship between baseline FVC (% predicted) and **(A)** CTG repeat size (*r* = −0.482, *p* = < 0.0001, *n* = 74, Pearson), **(B)** MIRS (*r* = −0.510, *p* < 0.0001, *n* = 100, Pearson), and **(C)** disease duration (*r* = −0.353, *p* = 0.0027, *n* = 70, Pearson).

In Table 1 the demographics, baseline characteristics, and duration of followup are presented for our population of 110 patients with DM1. Associations between baseline characteristics and respiratory dysfunction as measured by FVC (% predicted) were investigated. Greater CTG repeat sizes (*p* < 0.0001), MIRS Muscular Impairment Rate Scale (MIRS) scores (*p* < 0.0001), and disease duration (*p* = 0.0027) were associated with lower baseline FVC (% predicted) (Figure 1). Similarly, lower FVC (% predicted) was associated with higher BMI (*r* = −0.25, *p* < 0.001), presence of respiratory symptoms (any) (*r* = −0.20, *p* = < 0.001), and the presence of specific respiratory symptoms including orthopnea (orthopnea: median= 60.00 interquartile range= [48.00, 72.00], no orthopnea: 68.95 [57.00, 80.00] *p* = 0.008); shortness of breath (SOB: 64.9 [47.0,76.2], no SOB: 76.5 [60.4, 84.4], *p* = 0.0021), and dyspnea on exertion (DOE:69.2, [49.9, 79.0]), no DOE: 75.0 [60.1, 85.3], *p* = 0.038). Other specific respiratory symptoms including night sweats, morning headache, and daytime fatigue did not show significant associations with FVC at baseline. At baseline there was no relationship between number of respiratory symptoms and CTG repeat size and disease duration.

**Table 1:** Demographics of the 110 DM1 patients.

	***N* (%)**	**Mean ± SD**
**Sex**		
M	53 (47.7)	
W	57 (51.4)	
**Ethnicity**		
White/ European	99 (90.0)	
African American	5 (4.5)	
Hispanic/Latino	1 (0.9)	
Asian	1 (0.9)	
Other	1 (0.9)	
Unknown	3 (2.7)	
Age at baseline (years)		42.92 ± 12.34
BMI at baseline (kg/m^2^)	*n* = 107	27.05 ± 6.95
Age of onset	*n* = 70	29.29 ± 16.82
Congenital (apparent at birth)	3 (4.3)	
Childhood-onset (<10 years)	4 (5.7)	
Juvenile-onset (10–20)	15 (21.4)	
Adult-onset (20–40)	31(44.3)	
Late-onset (>40)	17 (24.3)	
Duration of disease at final collection point (years)	*n* = 70	18.63 ± 12.65
**CTG Expansion Score**		
1= (<100)	6 (8.1)	
2= (100–199)	13 (17.6)	
3= (200–699)	23 (31.1)	
4= (≥700)	32 (43.2)	
**Baseline MIRS Category**		
I	2 (1.8)	
II	25 (22.7)	
III	23 (20.9)	
IV	42 (38.2)	
V	8 (7.3)	
unknown	10 (9.1)	
Number of PFT evaluations		4.96 ± 3.02
Time from baseline to final PFT (years) evaluation		4.37 ± 3.01
PFT score at baseline (FVC % predicted)		
Normal (>79%)	23 (20.9)	
Mild restriction (70–79%)	32 (29.1)	
Moderate Restriction (50–69%)	36 (32.7)	
Severe Restriction (<50%)	19 (17.3)	
**Change in PFT Score from Baseline to Final Measurement**		
Never developed restrictive pattern	10 (9.3)	
Developed Restrictive pattern	13 (12.0)	
Started with restrictive pattern	85 (78.7)	

The primary parameter investigated in this study was FVC, but we also evaluated EtCO_2_ and VC (% predicted). At baseline there was no relationship between EtCO_2_ vs. CTG repeat size (*r* = 0.0314, *p* = 0.801, *n* = 67), MIRS score (*r* = 0.0481, *p* = 0.645, *n* = 94), or disease duration (*r* = 0.112, *p* = 0.385, *n* = 62). At baseline there was no relationship between VC (% predicted) and CTG repeat size (*r* = −0.452, *p* = 0.0790, *n* = 16), MIRS score (*r* = −0.443, *p* = 0.0985, *n* = 15), or disease duration (*r* = −0.0230, *p* = 0.941, *n* = 13).

### Longitudinal Decline in FVC Over Time and Predictive Factors

**Figure 2:**
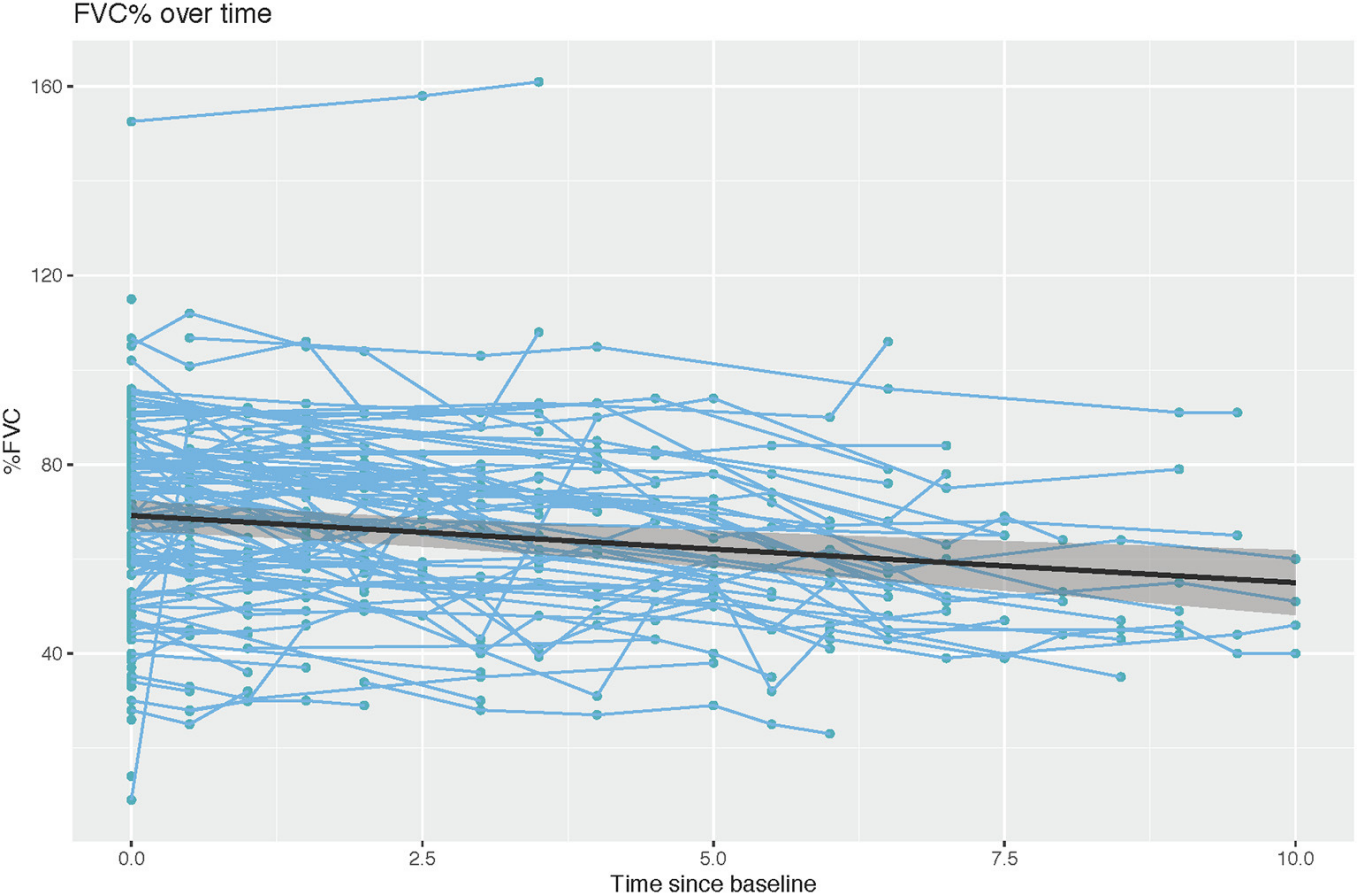
Rate of change in percent predicted FVC over time (*r* = −1.421, Wald statistic = 13.92 [*p* < 0.001], *n* = 152 subjects with 522 observations).

We analyzed the longitudinal FVC of the entire cohort to understand the progression of respiratory muscle dysfunction. The slope estimate for the unadjusted annual change of FVC (% predicted) obtained from generalized least squares was −1.42 (*p* < 0.001), indicating a negative linear trend between rate of change in FVC and time (Table 2, Figure 2).

**Table 2:** Longitudinal Decline in FVC over time.

	**Value**	**Std Error**	***t*-value**	***p*-value**
Intercept	69.2	1.65	42.1	0
Time since baseline PFTs (in years)	−1.42	0.381	−3.73	0

**Figure 3:**
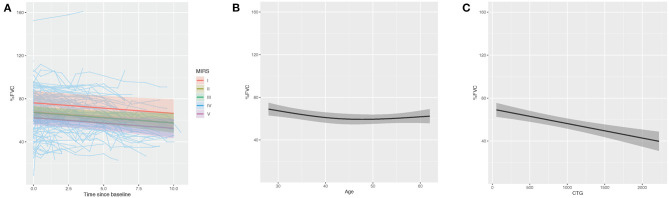
Multiple regression modeling of FVC (% predicted) across **(A)** MIRS, **(B)** age at time obtained and **(C)** CTG repeat length.

To understand what factors may be associated with rate of progression we analyzed FVC progression by adjusting skeletal muscle dysfunction as quantified by MIRS, CTG repeat length and time at time obtained as predictors of rate of decline. Longitudinal assessment demonstrated that patients with more severe muscle impairment at baseline measurement (MIRS IV and V) showed lower % predicted FVC than those with minimal impairment at baseline (MIRS I) (*p* = 0.012 and 0.025) and MIRS III (*p* = 0.011) (Table 3, Figure 3A). In patients with mild or moderate muscle impairment at baseline (MIRS II and III) % predicted FVC was not significantly different than the % predicted FVC of those with minimal impairment at baseline (MIRS I). There is a curvilinear correlation between % predicted FVC and age at time obtained (*p* = 0.008) (Table 3, Figure 3B). CTG repeat length showed negative correlation with % predicted FVC while controlling other covariates (*P* < 0.001) (Table 3, Figure 3C).

**Table 3:** Multiple regression of change in FVC (% predicted).

	**Value**	**Std.Error**	***t*-value**	***p*-value**
Intercept	117.4	11.2	10.440	0.000
Time since baseline PFTs (in years)	−0.902	0.500	−1.80	0.072
MIRS=II (ref: MIRS I)	−8.84	5.57	−1.58	0.113
MIRS=III (ref: MIRS I)	−8.78	5.67	−1.55	0.122
MIRS=IV (ref: MIRS I)	−13.9	5.51	−2.53	0.012
MIRS=V (ref: MIRS I)	−13.7	6.10	−2.25	0.025
MIRS= III (ref: MIRS IV & V)	5.090	1.983	2.566	0.011
CTG	−0.014	0.003	−4.78	0.000
Age at time obtained	−0.755	0.285	−2.65	0.008

### Annual Rate of Change of FVC and Predictive Factors

We calculated the annual rate of change in percent predicted FVC calculated by dividing the FVC change between the duration of two consecutive visits and associated it with factors including CTG repeat size, MIRS rating groups, age of symptoms onset and BMI. Rate of change of percent predicted FVC was not significantly different when compared across CTG repeat score (very small= 1.93 [−2.39, 3.67], small= −2.25 [−6.50, −0.00], medium= −1.22 [−4.38, 2.35], large= −1.67 [−6.00, 4.20], *p* = 0.27), MIRS rating groups (I= 2.08 [2.08, 2.08], II= −1.43 [−6.36, 2.22], III= −1.67 [−5.20, 1.96], IV= −1.46 [−5.60, 3.25], V= −0.87 [−4.94, 6.00], *p* = 0.92), age of symptom onset (congenital= −1.43 [−7.27, 2.00], childhood= −1.67 [−5.23, 2.40], juvenile= −1.67 [−5.50, 3.33], adult= −1.80 [−5.65, 2.22], late= −1.15 [−6.00, 3.33], *p* = 0.92), or BMI (r = −0.017, *p* = 0.69).

### Effect of Intervention

**Figure 4:**
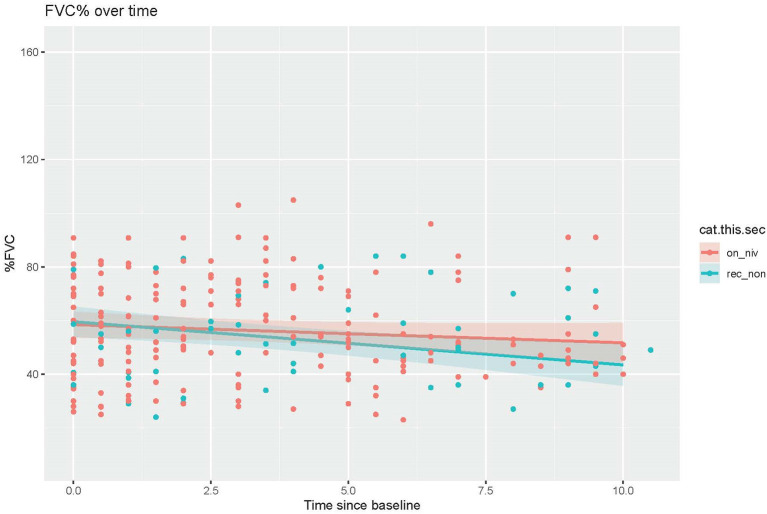
Comparison of decline in percent predicted FVC over time between patients recommended but non-compliant with NIV vs. those on NIV (*t* = −2.319, *p* = 0.021).

We analyzed the effect of NIV compliance among patients recommended to use NIV by comparing the change in percent predicted FVC between those who were and those who were not compliant with NIV. Patients who were recommended to use NIV but were non-compliant had percent predicted FVCs that decreased faster than those on NIV (NIV compliant= −0.68, NIV non-compliant= −1.61; *n* = 74; *p* = 0.021; Figure 4).

## Discussion

In this retrospective study, we aimed to better understand respiratory muscle impairment in patients with DM1. Respiratory impairment is an important feature of DM1 and is one of the most common muscular dystrophies associated with prominent respiratory failure. We found that FVC was impaired in the majority of individuals in our cohort. Similar to prior studies, we demonstrated that greater CTG repeat expansion and, accordingly, more severe muscle dysfunction, were associated with greater restrictive impairment (4, 6, 7, 13–15). We also found that respiratory muscle impairment was associated with increased patient symptom burden. At baseline, patients with more respiratory symptoms, such as shortness of breath, dyspnea on exertion, orthopnea, night sweats, morning headaches, and snoring, had lower FVC values. A similar association was seen in a recent study investigating the respiratory symptom burden in DM1 patients which found that the presence of more symptoms overall- orthopnea, dyspnea, apnea, poor sleep, morning headache, decreased concentration, daytime sleepiness, fatigue, and treated chest infections- correlated with worse pulmonary function as measured by PFTs (16). The association between orthopnea and lower baseline FVC is congruent with the established worsening of DM1 respiratory muscle function in supine positioning and further indicative of significant respiratory muscle weakness (4). Our findings of significant relationships between lower FVC and the presence of SOB, DOE, and orthopnea suggest that these symptoms may be more useful for screening as compared with symptoms of night sweats, morning headaches, and snoring. However, our study is limited by the lack of analysis of function of other organ systems, most notably the absence of attention to cardiac function as cardiac deficits can have significant overlap with the symptoms of respiratory dysfunction such as DOE and orthopnea.

Another major objective of this study was investigation of the rate of longitudinal respiratory muscle function decline in DM1. In our study, % predicted FVC showed a mean rate of decline of −1.42 % predicted FVC per year over an average period of 4.37 ± 3.01 years per patient. Importantly, in our study, we analyzed % predicted FVC as this factors in age, and allowed us account for the expected reductions of FVC during aging. When examined across all timepoints, there was a break from a pattern of progressive decline in that a curvilinear pattern between FVC and age was noted. FVC values were highest in the 2nd−3rd decade, reached a nadir in the 4th– 5th decade, and then slightly increased in the 5th−7th decade. This pattern may be explained by survivorship bias- those patients who survive past the 5th decade are likely have milder disease and thus show less severe respiratory impairment. The annual rate of % predicted FVC decline in our study was increased compared with that previously noted by Thil and colleagues who showed a rate of −0.72 predicted per year over an average of 9.02 ± 3.4 years per patient (8). It is possible that the greater rate of decline seen in our study may be explained by the increased severity of our cohort as only 9% of our patient population, as compared with 35% of the cohort examined by Thil et al. (8) showed no impairment of FVC.

This study attempted to identify factors that may be predictive of the rate of decline in respiratory function. We found no relationship between the rate of decline FVC when stratified by MIRS, CTG repeat length, age of onset, or BMI. As expected we did find that patients with more severe muscle impairment (baseline MIRS IV and V) demonstrated lower FVC than those with baseline MIRS I and III, but the rate of decline was not different between patients with differing baseline severities of muscle impairment. Factors such as BMI have been reported as an important factor in respiratory muscle dysfunction, and thus the lack of relationships between rate of respiratory decline and MIRS rating groups, CTG repeat length, or BMI was unexpected (17). This lack of significant differences in rates of FVC decline between MIRS rating groups may suggest that decline is not due to progressive muscle impairment alone (18). The associations with greater muscle impairment and worse respiratory function but no differences in rate of progression may suggest impairment due to developmental aspects of DM1. As such, more severely impaired patients may show more impairment at baseline, due to developmental defects, but similar rates of decline.

We examined various parameters of respiratory functional status, and we found FVC, as compared with EtCO_2_ or VC to be most correlated with other measures of disease severity. Thus, FVC was used to investigate predictors of decline of respiratory muscle function. One limitation of our study was that analyses of VC relative to other disease measured was limited by lack of baseline data for VC in 93 of our 110 participants as VC did not become a routine part of spirometry for our DM1 patients until a few years after our data collection starting point.

Other studies have commonly investigated FVC and found it to be impaired to varying degrees and some have observed both its variation with disease parameters such as MIRS score or CTG repeat length and its decline with disease progression (6, 8, 10, 14, 15). In a recent review of 55 articles on respiratory dysfunction in DM1 by Hawkins et al. (6) FVC and VC were the most commonly impaired parameters, with 23 of these articles utilizing FVC in their evaluation of respiratory dysfunction. Poussel et al. found a negative correlation between FVC (% predicted) and CTG repeat length, while Monteiro et al. found a correlation between CTG repeat length and SpO_2_, MEP (% predicted), and NIV use but not FVC (7, 15). Kierkegaard et al. found more severe respiratory function in patients with more severe muscle impairment, and similarly Araujo et al. found loss of expiratory muscle strength with increasing MIRS score (19, 20).

Data on the benefit of NIV use in DM1 is inconclusive. While Montiero et al. found that prolonged NIV use improved symptoms and improved nocturnal ventilation, other studies have demonstrated little benefit in symptoms or quality of life, as well as uncertain survival benefit for DM1 patients using NIV (7, 18). Here, we explored the impact of NIV compliance on the rate of decline of percent predicted FVC in patients recommended to receive NIV and found that NIV compliant patients experiences slower rates of FVC decline than non-compliant patients. These results support the utility of NIV intervention in slowing the progression of respiratory decline in DM1 patients. As a multisystem disease with diverse impact, the factors driving non-compliance, such as cognitive impairment or apathy, may also contribute to progression. Thus, it is difficult to conclude whether NIV directly contributed to less decline in the compliant individuals in this cohort.

The main strength of our study is longitudinal data obtained from a large cohort of DM1 patients showing slowly progressive respiratory decline. Our study was limited by wide variability both overall and within cohorts making effective comparison between these cohorts challenging. Despite comparing decline in multiple respiratory parameters across multiple disease measures we were unable to predict the cause of this variability. Interrater factors were controlled for by the use of a single respiratory therapist for all PFT measurements but there is still high likelihood that effort and compliance varied both between patients and visits. The ethnic population of our study poorly approximates the local ethnic composition, which may be a component of both founder effect and ascertainment issues possibly confounded by issues with unequal access to care. Another area of limitation for our study is related to its retrospective design not allowing for availability of all data across study duration, as with the lack of VC data. A possible area of future study is relation of respiratory decline to other system dysfunction, such as cardiac or endocrine dysfunction.

## Conclusion

This study confirms the current consensus that DM1 is characterized by slowly progressive restrictive respiratory dysfunction. We found FVC (% predicted) to be most correlated with other measures of disease severity, suggesting its utility as a measure of impairment of respiratory function in DM1. Greater CTG repeat size, higher MIRS rating, longer disease duration, and number of respiratory symptoms were all correlated with lower baseline FVC (% predicted), but annual rate of change was not correlated with any of these disease measures. The confirmation of progressive respiratory dysfunction and data suggesting less severe FVC in individuals that were compliant with NIV support the relevance of NIV treatment in combination with other therapeutic interventions to reduce respiratory decline in DM1. It is clear that there is a complex interplay of factors including but not limited to muscle weakness, genetic phenotype, age of onset, and BMI that are contributing to the progression of respiratory decline.

## Data Availability Statement

The raw data supporting the conclusions of this article will be made available by the authors, without undue reservation.

## Ethics Statement

The studies involving human participants were reviewed and approved by The Ohio State University. Written informed consent for participation was not required for this study in accordance with the national legislation and the institutional requirements.

## Author Contributions

WA and SL designed the study. LH, JZ, JR, GB, FV, WA, and SL collected and analyzed the data. LH, JZ, WA, and SL interpreted the results. LH drafted the manuscript. JZ, JR, GB, FV, WA, and SL were major contributors in writing and editing the manuscript. All authors read, revised, and approved the final manuscript.

## Conflict of Interest

The authors declare that the research was conducted in the absence of any commercial or financial relationships that could be construed as a potential conflict of interest.

## References

[1] Emery AEH. Population frequencies of inherited neuromuscular diseases-a world survey. Neuromuscul Disord. (1991) 1:19–29. 10.1016/0960-8966(91)90039-U 1822774

[2] LoRusso S Weiner B Arnold WD. Myotonic dystrophies: targeting therapies for multisystem disease. Neurotherapeutics. (2018) 15:872–84. 10.1007/s13311-018-00679-z 30341596 PMC6277298

[3] Mathieu J Allard P Potvin L Prevost C Begin P. A 10-year study of mortality in a cohort of patients with myotonic dystrophy. Neurology. (1999) 52:1658–62. 10.1212/WNL.52.8.1658 10331695

[4] Poussel M Kaminsky P Renaud P Laroppe J Pruna L Chenuel B. Supine changes in lung function correlate with chronic respiratory failure in myotonic dystrophy patients. Respir Physiol Neurobiol. (2014) 193:43–51. 10.1016/j.resp.2014.01.006 24440340

[5] Pfeffer G Povitz M Gibson GJ Chinnery PF. Diagnosis of muscle diseases presenting with early respiratory failure. J Neurol. (2015) 5:1101–14. 10.1007/s00415-014-7526-1 25377282

[6] Hawkins AM Hawkins CL Abdul Razak K Khoo TK Tran K Jackson RV. Respiratory dysfunction in myotonic dystrophy type 1: a systematic review. Neuromuscul Disord. (2019) 29:198–212. 10.1016/j.nmd.2018.12.002 30765255

[7] Monteiro R Bento J Goncalves MR Pinto T Winck JC. Genetics correlates with lung function and nocturnal ventilation in myotonic dystrophy. Sleep Breath. (2013) 17:1087–92. 10.1007/s11325-013-0807-6 23319325

[8] Thil C Agrinier N Chenuel B Poussel M. Longitudinal course of lung function in myotonic dystrophy type 1. Muscle Nerve. (2017) 56:816–8. 10.1002/mus.25604 28181267

[9] Igo M. [Serial observations of respiratory function in disabled patients with myotonic dystrophy]. Rinsho Shinkeigaku. (1993) 33:845–9. 8261695

[10] Nitz JC Burns YR Jackson RV. A longitudinal physical profile assessment of skeletal muscle manifestations in myotonic dystrophy. Clin Rehabil. (1999) 13:64–73. 10.1177/026921559901300109 10327099

[11] Dahlbom K Ahlström G Barany M Kihlgren A Gunnarsson LG. Muscular dystrophy in adults: a five-year follow-up. Scand J Rehabil Med. (1999) 31:178–84. 10.1080/003655099444524 10458316

[12] Mathieu J Boivin H Meunier D Gaudreault M Bégin P. Assessment of a disease-specific muscular impairment rating scale in myotonic dystrophy. Neurology. (2001) 56:336–40. 10.1212/WNL.56.3.336 11171898

[13] Vivekananda U Turner C. A model to predict ventilator requirement in myotonic dystrophy type 1. Muscle Nerve. (2019) 59:683–7. 10.1002/mus.26471 30895625

[14] Rossi S Della Marca G Ricci M Perna A Nicoletti TF Brunetti V. Prevalence and predictor factors of respiratory impairment in a large cohort of patients with Myotonic Dystrophy type 1 (DM1): a retrospective, cross sectional study. J Neurol Sci. (2019) 399:118–24. 10.1016/j.jns.2019.02.012 30798109

[15] Poussel M Thil C Kaminsky P Mercy M Gomez E Chaouat A. Lack of correlation between the ventilatory response to CO2 and lung function impairment in myotonic dystrophy patients: evidence for a dysregulation at central level. Neuromuscul Disord. (2015) 25:403–8. 10.1016/j.nmd.2015.02.006 25753091

[16] De Mattia E Lizio A Falcier E Sannicolò G Gualandris M Rossi G. Screening for early symptoms of respiratory involvement in myotonic dystrophy type 1 using the Respicheck questionnaire. Neuromuscul Disord. (2020) 30:301–9. 10.1016/j.nmd.2020.02.014 32305258

[17] Seijger CG Drost G Posma JM van Engelen BG Heijdra YF. Overweight is an independent risk factor for reduced lung volumes in myotonic dystrophy type 1. PLoS ONE. (2016) 11:e0152344. 10.1371/journal.pone.0152344 27015655 PMC4807837

[18] Serisier DE Mastaglia FL Gibson GJ. Respiratory muscle function and ventilatory control. I in patients with motor neurone disease. II in patients with myotonic dystrophy. Q J Med. (1982) 51:205–26. 6810404

[19] Kierkegaard M Harms-Ringdahl K Holmqvist LW Tollbäck A. Functioning and disability in adults with myotonic dystrophy type 1. Disabil Rehabil. (2011) 33:1826–36. 10.3109/09638288.2010.549287 21254917

[20] Araújo TL Resqueti VR Bruno S Azevedo IG Dourado ME Jr Fregonezi G. Respiratory muscle strength and quality of life in myotonic dystrophy patients. Rev Port Pneumol. (2010) 16:892–8. 10.1016/S2173-5115(10)70006-3 21067695

